# Initial Experiences of Selective RET Inhibitor Selpercatinib in Adults with Metastatic Differentiated Thyroid Carcinoma and Medullary Thyroid Carcinoma: Real-World Case Series in Korea

**DOI:** 10.3390/curroncol30030229

**Published:** 2023-03-03

**Authors:** Han-Sang Baek, Jeonghoon Ha, Seunggyun Ha, Ja Seong Bae, Chan Kwon Jung, Dong-Jun Lim

**Affiliations:** 1 Division of Endocrinology and Metabolism, Department of Internal Medicine, Seoul St. Mary’s Hospital, College of Medicine, The Catholic University of Korea, 222, Banpo-daero, Seocho-gu, Seoul 06591, Republic of Korea; 2 Division of Nuclear Medicine, Department of Radiology, Seoul St. Mary’s Hospital, College of Medicine, The Catholic University of Korea, 222, Banpo-daero, Seocho-gu, Seoul 06591, Republic of Korea; 3 Department of Surgery, College of Medicine, The Catholic University of Korea, 222, Banpo-daero, Seocho-gu, Seoul 06591, Republic of Korea; 4 Department of Hospital Pathology, College of Medicine, The Catholic University of Korea, 222, Banpo-daero, Seocho-gu, Seoul 06591, Republic of Korea

**Keywords:** selpercatinib, *RET* alteration, thyroid cancer, papillary, thyroid cancer, medullary, lenvatinib, gastrostomy

## Abstract

**Simple Summary:**

Recently, selpercatinib, a highly selective inhibitor of *RET* receptor tyrosine kinase, has been used for *RET*-altered thyroid cancer. However, real-world data of its effectiveness and safety in various clinical situations are lacking. We present four cases of patients with advanced thyroid cancer who were treated with selpercatinib with variable clinical situations. Selpercatinib showed good efficacy in all four patients without significant side effects. Despite facing varying clinical obstacles of the real world, selpercatinib safely proved remarkable therapeutic efficacy, although drug safety and durability through long-term use should be further validated.

**Abstract:**

Recently, selpercatinib, a highly selective inhibitor of *RET* receptor tyrosine kinase, has been used for *RET*-altered thyroid cancer. We present four cases of patients with advanced thyroid cancer who were treated with selpercatinib. The first patient was a 63-year-old male with advanced medullary thyroid cancer (MTC) treated with vandetanib. Six months ago, he had an intracranial hemorrhage and swallowing difficulty. He started selpercatinib with percutaneous endoscopic gastrostomy (PEG). For 11 months, a partial response (PR) was observed stably with PEG administration without any more cardiovascular events. The second patient was a 67-year-old female with advanced MTC treated with vandetatib. After selpercatinib treatment, a PR was observed for most metastatic sites, including choroidal metastasis. The third patient was a 32-year-old female with advanced papillary thyroid cancer (PTC) without history of systematic treatment. For six months, a PR was observed at her metastatic site with manageable adverse events. The last patient was a 59-year-old female with advanced PTC treated with lenvatinib. She suffered from a panic disorder and pleural pain due to metastasis during lenvatinib treatment. After selpercatinib treatment, her pain and panic symptoms were improved. Facing varying clinical obstacles of the real world, selpercatinib safely proved remarkable therapeutic efficacy regardless of previous treatment or metastatic site.

## 1. Introduction

*RET* receptor tyrosine kinase can be activated as proto-oncogene through two major mechanisms: point mutation and gene fusion [1,2,3]. *RET* mutation is associated with most cases of medullary thyroid cancers (MTCs) [4]. Germline *RET* mutation is associated with hereditary multiple endocrine neoplasia (MEN). It accounts for 25% of MTC cases. The remaining 75% of MTC cases are sporadic MTC, of which about 60% have somatic *RET* mutations [1]. *RET* mutation is known to be a risk factor for aggressive disease, and many patients with metastatic MTC harbor *RET* mutations [5]. Meanwhile, *RET* fusion occurs in many kinds of cancers, including 1~2% of lung cancers and about 10% of differentiated thyroid cancers, but it rarely occurs in other solid tumors [1,2]. *RET* fusion is highly detected in young patients (20–60%) and radiation-associated thyroid cancers [1,6].

Multitargeted kinase inhibitors (MKI), such as vandetanib and cabozantinib, for MTC and lenvatinib and sorafenib for papillary thyroid cancer (PTC), have been used as first-line treatments for advanced thyroid cancer [7,8,9,10,11]. These MKIs could also be used for *RET*-altered malignancies. However, these nonselective *RET* inhibitors show only modest efficacy with limited use due to adverse events because they predominantly inhibit non-*RET* kinases in conjunction with weak anti-*RET* specific inhibition. These off-target side-effects could be the reasons for their frequent dose reductions [1,2].

Selpercatinib, an ATP-competitive, highly selective, small-molecule inhibitor for *RET*, has been approved by the Food and Drug Administration (FDA) for *RET*-mutant cancers [12]. In a Phase 1–2 trial, the response rate for RET-mutant MTC was 69% in patients who previously received MKI treatment and 73% in patients who previously did not receive MKI treatment [1]. For *RET* fusion-positive thyroid cancer, the response rate was 79% with low adverse events [1]. Selpercatinib also showed good efficacy in studies using a Chinese population [13]. It has been suggested that other *RET* alterations with less frequency, such as *indels* which indicate complex alterations such as small deletions or insertions or both, can also be treated with selpercatnib [14].

However, real-world data on the effectiveness and safety of selpercatinib in various clinical situations, such as enteral administration of the drug via PEG tube, troublesome malignant pleural effusions, and arrhythmia as an adverse event, are lacking. Therefore, the objective of this study was to describe four real-world cases of patients with advanced thyroid cancer treated with selpercatinib.

## 2. Methods

Data used in this study were approved by the Catholic University Data Review Committee. All data were anonymized. Due to the nature of a retrospective study, there was no possibility of physical or mental harm to patients as a result of this study. Therefore, the requirement for informed consent was waived by the Institutional Review Board (IRB). This study adhered to the tenets of the Declaration of Helsinki. It was approved by the Institutional Review Board of the Catholic University of Korea (KC22RISI0975).

The patient’s disease status was assessed using thyroid ultrasonography, computed tomography (CT) scan, bone scan, and 18-fluorodeoxyglucose (FDG) positron emission tomography-CT (PET-CT). The Response Evaluation Criteria in Solid Tumors (RECIST) 1.1 criteria were used to evaluate responses to treatments [15]. The RECIST criteria were defined as follows: complete response (CR), disappearance of all targets; partial response (PR), decrease in the number of metastatic nodules or 30% decrease in the sum of the longest diameter of target lesions; progressive disease (PD), increased number of metastatic nodules or 20% increase in the sum of the longest diameter of target lesions; and stable disease (SD), neither progression nor regression. Adverse effects were graded 1 to 4 according to the Common Terminology Criteria for Adverse Events (CTCAE): Grade 1, mild; Grade 2, moderate; Grade 3, severe; and Grade 4, life-threatening.

### 2.1. Case 1

**Figure 1:**
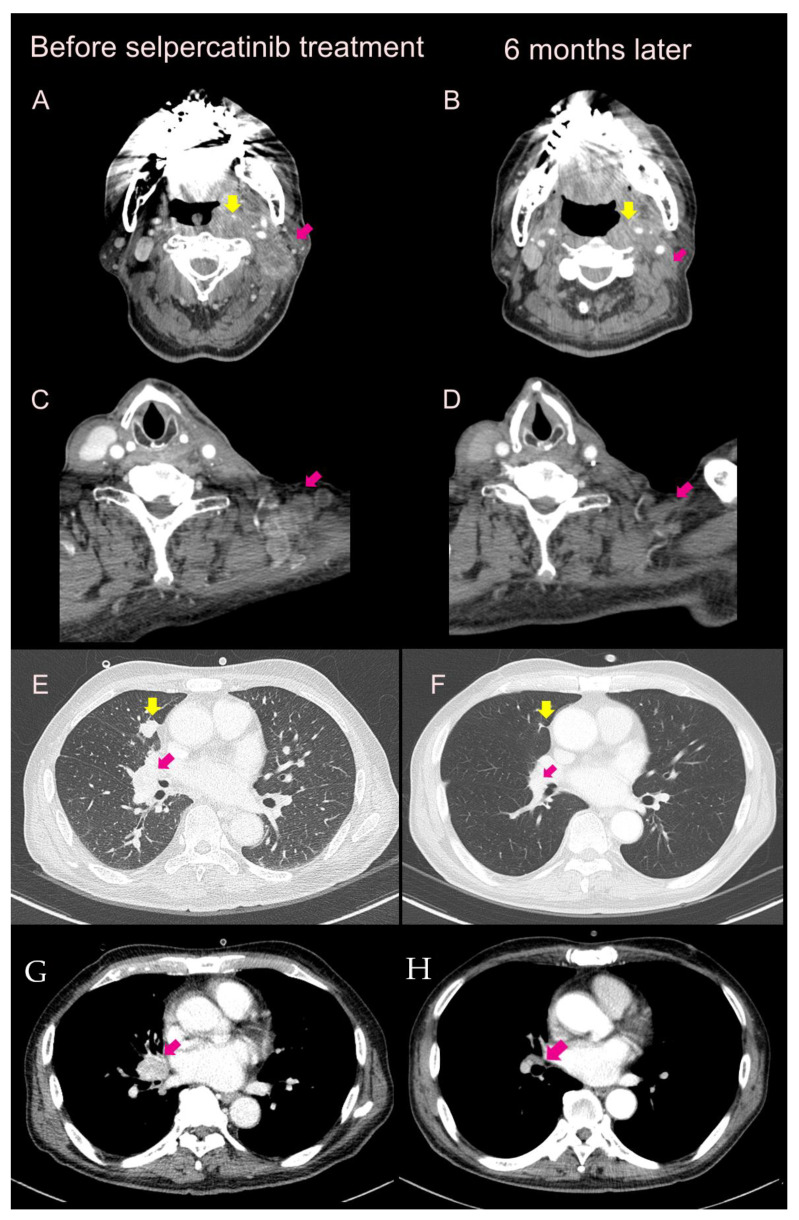
CT scan images of the Case 1 patient before and at six months after selpercatinib treatment. Before selpercatinib treatment, there were metastatic lesions at the neck (**A**); lateral lymph node (**C**); lung parenchym (**E**); and hilar lesion (**G**). All of them showed regression at six months after selpercatinib treatment (**B**,**D**,**F**,**H**) (yellow and purple arrows).

A 62-year-old male patient visited the outpatient clinic for a second-line tyrosine kinase inhibitor (TKI) treatment for advanced MTC. The patient underwent total thyroidectomy (TT) with both modified radical neck dissection (MRND) for MTC six years before. He also received double wedge resection of the right middle lobe (RML) nodule and suspicious mediastinal lymph node (LN) biopsy by video-assisted thoracic surgery (VATS). The TNM stage was T3N1bMx according to the Seventh American Joint Committee on Cancer (AJCC) stage system. The tumor size from the VATS resection was 0.6 × 0.5 cm at the RML site confirmed as metastatic MTC. As MTC was diagnosed, a mutation test was performed using a blood sample, which confirmed *RET* germline mutation (exon15; c.2692G > T[D898Y]). As the lung metastatic lesion showed progression on the follow-up CT scan, the patient was started on vandetanib treatment with an initial dose of 300 mg daily. However, due to Grade 3 QT corrected (QTc) interval prolongation in an electrocardiogram (ECG), he could not maintain the initial dose and used a reduced dose (200 mg daily). The patient showed the best response at 22 months as partial response. After about 30 months of treatment, multiple bone metastases were newly discovered and diagnosed as progressive disease (PD), so vandetanib treatment was discontinued. At that time, palliative radiation therapy (RT) was performed for multiple spine metastasis, and there was no second-line TKI available in Korea at that time. One year after suspension of vandetanib, the patient was admitted to the emergency room (ER) with a syncope and decreased mentality. Intracranial hemorrhage (ICH) was confirmed with a brain CT performed in the ER. After intensive care unit (ICU) care, his mentality was restored, but he continued to experience difficulty swallowing, leading to a percutaneous endoscopic gastrostomy (PEG) procedure. Meanwhile, metastatic lymphadenopathy of the neck causing pain and progression of multiple bone metastases were confirmed. With palliative radiation therapy (RT) on the neck lesion, selpercatinib treatment was prepared and started. After six months of administration of selpercatinib, a partial response on a CT scan was observed, and the patient’s pain from the neck lesion was relieved (Figure 1). Furthermore, a bone scan, multiple bone metastases in the sternum, cervical-thoracic spines, and bilateral ribs showed markedly regressed activity suggesting partial response. To administer the medication using PEG, selpercatinib capsules were dissolved in warm water by stirring them in the water, melting the capsule shell, and then used.

**Figure 2:**
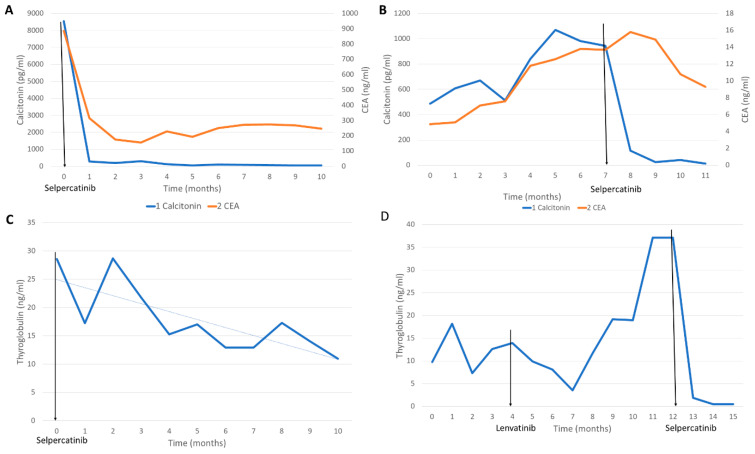
The changes of tumor markers during the selpercatinib treatment for each patient: (**A**) the changes of calcitonin and carcinoembryonic antigen (CEA) of the patient in Case 1; the levels were decreased (for calcitonin, from 8541 pg/mL to 52.91 pg/mL and for CEA, from 886 ng/mL to 247 ng/mL); (**B**) the calcitonin and CEA levels of the patient in Case 2 (for calcitonin, from 944.6 ng/mL to 12.19 ng/mL and for CEA, from 13.7 ng/mL to 9.32 ng/mL); (**C**) the thyroglobulin level of the patient in Case 3 (from 28.57 ng/mL to 10.96 ng/mL); (**D**) the thyroglobulin level of the patient in Case 4 (from 37.11 ng/mL to 0.48 ng/mL).

His blood calcitonin and carcinoembryonic antigen (CEA) levels were remarkably decreased. (For calcitonin, from 8541 pg/mL to 52.91 pg/mL and for CEA, from 886 ng/mL to 247 ng/mL) (Figure 2A).

Considering a history of ICH, a low dose of selpercatnib was started (120 mg twice a day). QTc prolongation did not worsen with selpercatinib administration (460 ms). The patient′s blood pressure and heart rate were monitored and controlled with valsartan 40 mg once daily and bisoprolol 2.5 mg daily, respectively, under the consultation of the cardiovascular (CV) department. At 12 weeks after using selpercatinib, hypercalcemia (13.4 mg/dL) and decreased estimated glomerular filtration rate (eGFR) (34 mL/min/1.73 m^2^) were confirmed as lab findings, and he was hospitalized. After stopping selpercatinib for one week and reducing calcium/vitamin D dose with conservative care, including hydration, the patient quickly recovered. Upon restarting the previous dose of selpercatinib, there were no further hypercalcemia events. Therefore, hypercalcemia is not clearly associated with selpercatinib. Partial response has been achieved for 11 months. Until now (about 1-year of treatment), his disease is stable with PEG administration of selpercatinib without any more CV events (Table 1).

### 2.2. Case 2

**Figure 3:**
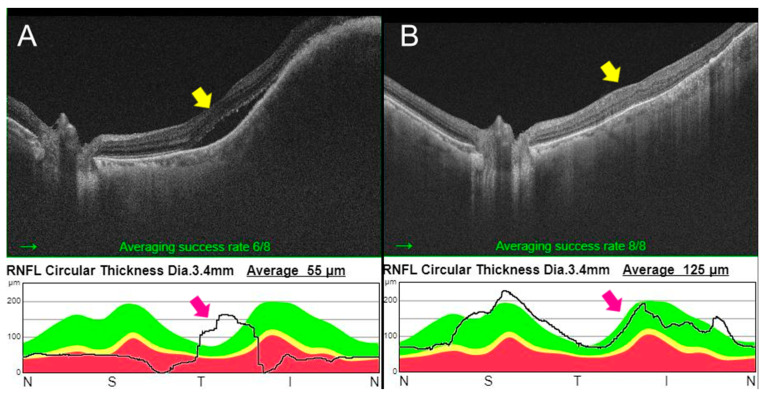
Optical coherence tomography (OCT) of left eye in Case 2 before and at one month after selpercatinib treatment; Image (**A**), before treatment; Image (**B**), after treatment; thickening of choroidal layers with irregular anterior surface is a typical feature of choroidal metastasis at OCT. It is well observed in (**A**) (yellow arrow) but not observed in (**B**). The thickness of each layer was out of normal range before treatment. It was improved after treatment (purple arrow, the black line).

A 67-year-old female came to the hospital to consult about secondary treatment for advanced medullary thyroid cancer. The patient underwent TT with central and lateral neck LN dissection for MTC 7 years before. The TNM stage was T3N1bMx according to the Seventh AJCC staging system. The RET germline mutation testing was offered due to confirmation of histology of MTC, but the germline RET mutation was not detectable. The patient was started on vandetanib 300 mg daily as first-line treatment but had to discontinue 24 months later due to Grade 3 QTc prolongation and Grade 2 vision blurring. In preparation for second line therapy, disease progression was detected in multiple LNs in PET-CT. At the same time, hepatic and multiple bone metastases were detected. In particular, since metastatic liver lesions were first detected, a liver biopsy confirmed metastatic MTC accompanied by *RET* mutation (M918T). At the same time, she complained of blurred vision. An ophthalmologic examination confirmed choroidal metastasis. Trans-arterial embolization (TAE) was performed twice on the metastatic liver lesion along with RT for spine and retina lesions. As the patient’s body weight was below 50 kg (47.8 kg), selpercatinib was initiated at a dose of 120 mg twice per day according to treatment guides provided by the pharmaceutical company [16]. At one month after selpercatinib treatment, a PR was seen in the choroidal lesion (Figure 3). On a CT scan, decreased size of paraaortic and retrocrural LNs was observed. Further decreased size of the TAE-treated metastatic lesion was also observed. Overall, a PR was observed in the abdomen and chest CT, and SD was observed in the neck CT.

The calcitonin (from 944.6 ng/mL to 12.19 ng/mL) and CEA (from 13.7 ng/mL to 9.32 ng/mL) levels were also improved (Figure 2B). QTc prolongation also improved as the accompanying hypothyroidism improved. The QTc prolongation was manageable with a beta-blocker under consultation of the cardiology division (Table 1).

### 2.3. Case 3

A 32-year-old female came to the hospital for treatment for advanced PTC. The patient underwent allogeneic bone marrow transplantation (allo-BMT) for acute myeloid leukemia (AML) at the age of 18, with a current hematologic disease-free condition. Eight years ago, for newly diagnosed PTC with multiple neck LN metastases, TT with central lymph node dissection, bilateral tracheal wall shaving, and left recurrent laryngeal LN shaving were performed. The largest tumor size was 4.8 × 2.7 × 1.9 cm in the left thyroid lobe, and metastasis to the cervical LN was found in the right LN level 3.4,5 and in the central, level 2,3,4,5 left side. The histological type was diffuse sclerosing type of PTC. The TNM stage at the time of surgery was T4aN1bMx according to the Seventh AJCC staging system. For multiple recurrent metastatic lesions in the neck and lung, repeated high dose RAI (totally, 300 mCi) and repeated radio-frequent ablation (RFA) were performed. Despite aggressive surgical treatment, RAI refractory disease was confirmed because there was no uptake in a post-treatment scan of multiple lung metastasis.

**Figure 4:**
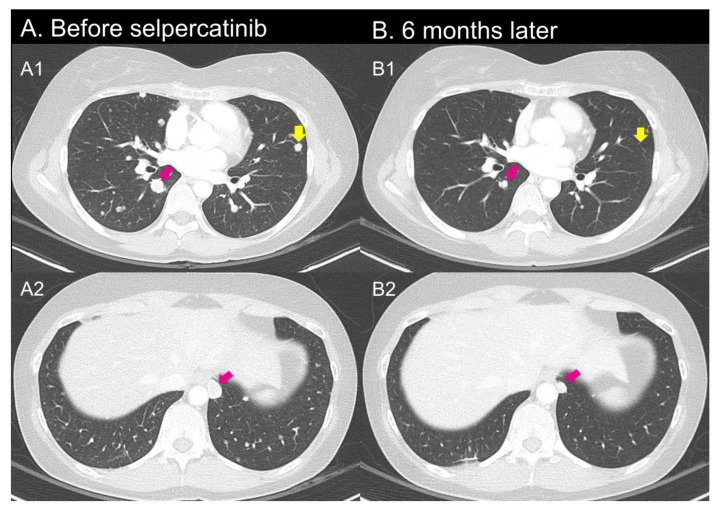
Before and at one month after selpercatinib treatment CT-scan of the patient in Case 3. Before treatment, there were metastatic lesions at hilar lesion, pleural, and lung parenchyme (**A1**,**A2**). After the treatment, all of them showed regression (yellow and purple arrows).

NGS on the tracheal metastatic lesion showed *CCDC6::RET* gene fusion. In fear of adverse events of VEGFR-based first-line TKI, she decided to use selective *RET* inhibitor instead of approved sorafenib or lenvatinib. For disease progression of multiple lung metastasis in the follow-up CT scan, selpercatinib (160 mg twice a day) treatment was started. At 6 months after treatment, the CT scan showed a partial response (PR) with a biochemically reduced thyroglobulin level (Figure 4).

The thyroglobulin level was gradually decreased during the treatment (from 28.57 ng/mL to 10.96 ng/mL) (Figure 2C).

As for her adverse events on selpercatinib, hypertension (HTN) was observed at three weeks after starting the treatment. Blood pressure was controlled to be below 130/80 by the administration of nifedipine 30 mg per day and olmesartan 20 mg per day. At 8 weeks after treatment, she complained of dry mouth, mild constipation, and headache. The severity of all symptoms was Grade 1. At 12 weeks after treatment, persisting dry mouth was successfully improved by pilocarpine. Skin photosensitivity, a frequent adverse event of *RET* inhibitor, was relieved by antihistamine. She complained of watery diarrhea of Grade 1 at 6 months after treatment. However, it was manageable with general antidiarrheal medications (Table 1).

### 2.4. Case 4

**Figure 5:**
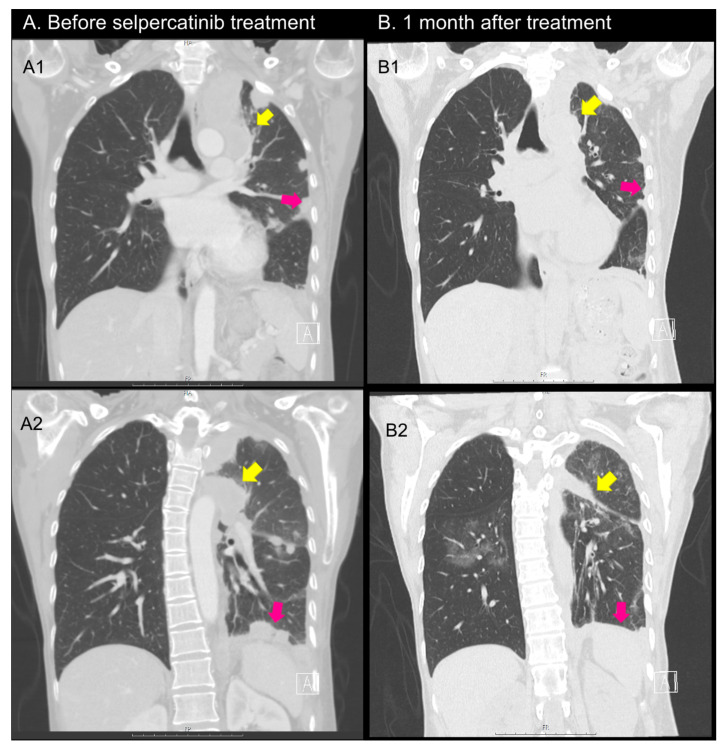
Before and at one month after selpercatinib treatment CT-scan of the patient in Case 4. Before treatment, there were metastatic lesions at hilar lesion, pleural, and lung parenchyme (**A1**,**A2**). After the treatment, all of them showed regression (**B1**,**B2**, yellow and purple arrows).

A 59-year-old female patient came to the hospital to discuss second-line TKI treatment for advanced PTC. She underwent TT 17 years ago with an RAI treatment of 150 mCi. The patient underwent an initial surgery at another hospital and was transferred to our hospital. Detailed pathology findings from the initial surgery are currently unknown. Six years after surgery, neck skin and sternal metastases (tumor size 1.8 cm) were confirmed. Thus, surgery and repeated RAI treatment (totally, 500 mCi) were performed for metastatic lesions. In a post-treatment whole-body scan, some lesions of the lung showed uptake, although some lesions did not, indicating radioiodine refractoriness. Two years later, she was admitted to the ER with dyspnea. Bilateral lung metastatic nodules and left malignant pleural effusion were confirmed on a CT scan. Pleural effusion drainage and VATS-assisted hyperthermia were performed. As a first-line TKI therapy, lenvatinib (20 mg per day) was started. However, she complained of poor oral intake and generalized pain. So, she could not maintain the initial dose. She repeated hospitalization and discharge due to uncontrolled malignant pleural effusion and adverse events of lenvatinib. In addition, she has been taking medication for a panic disorder. With lenvatinib treatment, her panic symptom worsened, which lowered the drug compliance. A whole-body PET-CT scan showed progressive and extensive metastatic tumors in lung, pleura, ribs, iliac bones, and pericardiac and mediastinal LNs. NGS with lung biopsy confirmed *CCDC6:RET* fusion. One month after selpercatinib 120 mg twice a day, CT scan showed a PR, and her dyspnea, chest pain, and anxiety symptoms dramatically improved with reduced pleural effusion (Figure 5).

The thyroglobulin level was also improved (from 37.11 ng/mL to 0.48 ng/mL) (Figure 2D). She only complained of mild diarrhea and dry mouth (Grade 1). Her follow-up ECG detected a few newly developed VPCs without QTc prolongation. As she had no symptoms related to arrhythmia, she continued to take selpercatinib without dose modification under close monitoring of a cardiologist. (Table 1).

## 3. Discussion

In Cases 1 and 2 of MTC with *RET* mutation and in Cases 3 and 4 of PTC with *RET*-fusion, selpercatinib treatment showed good therapeutic efficacy with tolerable and minor adverse events. As none of the four patients was involved in a clinical trial, our cases reflect the efficacy and tolerability of selpercatinib in various clinical situations of real-world practice. In a Phase 1–2 trial of selpercatinib, the objective response rate was 69% in 55 MTC patients who had previously received other MKI treatments and 73% in 88 patients who had not previously received MKI treatment. In 19 previously treated *RET* fusion-positive differentiated thyroid cancer patients, the objective response rate was 79% [1]. Responses were observed across all *RET* mutations. In this trial, *RET* M918T mutation for MTC and *CCDC6::RET* fusion for RET-fusion positive PTC were the most common *RET* alterations.

The patient in Case 2 harbored an M918T mutation. This alteration was confirmed by NGS using tissues obtained from a metastatic hepatic nodule as the germline mutation was not detected in an examination with a blood sample at her early disease course. On the other hand, the germline *RET* mutation was confirmed in the patient of Case 1 with a blood sample. To the best of our knowledge, the *RET* D898Y mutation has not been reported in other references, and there is no information about its association with MTC. However, clinical constellations presented in Case 1 definitely showed a pathologic and poor prognostic role of the *RET* D898Y mutation. There is only one case report demonstrating unilateral pheochromocytoma with an *RET* D898Y mutation [17]. Although these two *RET* alterations were different from each other, both showed excellent therapeutic efficacy with more than a partial response to selpercatinib treatment.

PTC patients in Cases 3 and 4 both had *CCDC6::RET* fusion, one of the most common *RET* rearrangements among patients with PTC [18]. Both patients in our cases who had this mutation showed a good response to selpercatinib treatment. However, there have been no data of treatment outcome according to each genotype of the *RET* fusion gene. Further research should be conducted in the near future with a large population and real-world setting.

Although patients in our study had metastatic tumors in various organs, selpercatinib showed good therapeutic efficacy, leading to more than a PR within the first six months. Patients in our cases had many metastatic sites, including LN, lung, bone, and choroid in the eye. In particular, the patient in Case 2 with choroidal metastasis in our report also showed a significant response. In one case report, similar to our case, significant responses of MTC choroidal metastases to selpercatinib have been reported previously [19]. The LN metastasis of the patient in Case 1 showed a decrease in size with pain relief. Lung metastasis of the patient in Case 3 and pleural metastasis of the patient in Case 4 also showed improvement after the selpercatinib treatment.

Additionally, selpercatinib showed satisfactory efficacy even through PEG administration. In Case 1, we administrated selpercatinib via PEG. The feasibility and favorable outcomes of kinase inhibitors administration through feeding tubes or PEG in NSCLC patients have been reported [20]. NSCLC patients need medication through a nasogastric tube or PEG mostly because of respiratory impairment, requiring mechanical ventilation [20]. To the best of our knowledge, the present study is the first one that reports administration of selpercatinib through PEG. The patient showed good response to selpercatinib treatment without any remarkable adverse events.

Patients in Cases 1, 2, and 4 were previously treated by MKI before the selpercatinib treatment, whereas the patient in Case 3 used selpercatinib as first-line therapy. First-line use of a highly selective inhibitor targeting a specific mutation has not been established yet. It is different according to cancer type. However, treatment targeting a specific molecular alteration regardless of cancer type or previous treatment, a so-called tumor-agnostic drug, has been developed recently [21]. Selpercatinib also showed good treatment efficacy for patients with *RET* fusion-positive non-small cell lung cancer (NSCLC) or PTCs and *RET*-mutated MTC in the LIBRETTO-001 trial [21]. For treatment-naive NSCLC patients, the overall response rate (ORR) was 84%. For untreated MTC patients, the ORR was 73% with tolerable adverse events [1,22]. These facts provide a rationale for using selective kinase inhibitors as a first-line treatment.

*RET* gene alterations were confirmed through NGS in three of four cases. NGS could make it possible to obtain parallel sequencing of multiple genes rapidly. Thus, it could be helpful for differentiating thyroid cancer from a benign nodule and for identifying actionable targets in advanced thyroid cancer, emphasizing ‘precision medicine [23,24,25,26]. Although there is no clear guideline for application of NGS in advanced thyroid cancer, NGS could be useful for detecting the *RET* fusion gene [25,27,28].

Compared to MKI treatment, selpercatinib caused similar kinds of adverse events, which were less frequent and mostly manageable. One such adverse event that draws attention was QTc prolongation. In retrospective analysis of selpercatinib for NSCLC patients, 4% of patients experienced QTc prolongation of Grade 3 or higher [29]. In the selpercatinib clinical trial for thyroid cancer, 13% of patients experienced QTc interval prolonged, which was less frequent than in the vandetanib study [8]. In the vandetanib Phase 3 trial for MTC patients, more study subjects in the vandetanib group developed QTc prolongation compared to the placebo group (35% vs. 3%) [8]. Physicians should carefully monitor patients′ thyroid function, as hypothyroidism could be associated with QTc prolongation [30].

In addition, selpecatinib can be used safely in patients with underlying or on-going cardiovascular disease, different from other MKI treatments. MKIs have many cardiovascular off-target adverse effects because of their nonselective nature [18]. Typically, HTN is the class effect of drugs targeting the VEGF/VEGFR signaling pathway [31]. In Phase 3 trials, 76% of those receiving lenvatinib, 40.6% of those receiving sorafenib, and 32% of those receiving vandetanib experienced treatment-emergent HTN [8,9,31]. In a trial for *RET*-altered thyroid cancer, 21% of patients experienced Grade 3 or higher HTN [1]. The Case 1 patient took antihypertensive medication as treatment for adverse events during the first-line therapy of vandetanib. Afterwards, he experienced an intracranial hemorrhage. However, selpercatinib did not further aggravate his CV disease including hypertension, which was well-controlled with anti-hypertensive medications.

Psychiatric symptoms could be aggravated by treatment or adverse events of MKI therapy. In particular, VEGFR targeting could be the reason for psychiatric disorder worsening despite the role of a VEGF targeting agent in a psychiatric disorder is vague. There was a case report of two patients with renal cell carcinoma who experienced worsening of psychotic symptoms after sunitinib treatment [32]. The suggested mechanism was that reduced VEGF levels could influence synaptic activity and neuron development [32]. In another report, decreased cerebral blood flow by vasoconstriction associated with VEFGR-2 inhibition was proposed to be the underlying mechanism for neurological events after sunitinib administration in three patients [33]. In this respect, using selective inhibitors rather than VEGFR-based therapy could reduce adverse events such as psychiatric symptoms including panic disorder. The patient in Case 4 experienced worsening of a panic disorder during lenvatinib treatment. It was unclear whether the worsening of anxiety and panic disorder was due to adverse events of treatment or pain from disease progression. However, her panic symptoms have improved after selpercatinib administration without showing severe side effects.

The safety profile of selpercatinib was good; however, several emerging adverse treatment events are being observed, particularly as chylous effusions [34,35]. The monitoring of patients should be continued due to the relatively recent introduction of these drugs and the potential for late adverse events.

Facing varying clinical obstacles of the real world, such as drug administration via PEG, underlying recent cerebrovascular disease, and aggravation of a panic disorder by previous VEGFR-based MKI, selpercatinib safely proved remarkable therapeutic efficacy, although drug safety and durability through long-term use should be further validated.

## Figures and Tables

**Table 1:** Summary of cases.

	Case 1	Case 2	Case 3	Case 4
Age at selpercatinib treatment	62	67	32	59
Sex	M	F	F	F
Tumor type	MTC	MTC	PTC	PTC
Mutation	RET mutation D898Y Germline	RET mutation M918T Somatic	CCDC6-RET gene fusion	CCDC6-RET gene fusion
Previous TKI treatment	Vandetanib	Vandetanib	None	Lenvatinib
Detection methods for RET alteration	Germline mutation study on blood sample	NGS on hepatic metastatic tissue	NGS on neck LN metastatic tissue	NGS on lung metastatic tissue
Metastasis site	LN, lung, bone	LN, lung, liver, choroidal	LN, lung	LN, lung, pleura, bone
Adverse events with selpercatinib treatment ^a^	HTN (G3), QTc prolongation (G1), headache (G1~2)	QTc prolongation (G3)	HTN (G2), Dry mouth (G2), photosensitivity (G1), diarrhea (G1),	Dry mouth (G1) Diarrhea (G1) VPC (G1)
Key points of case	PEG administration History of ICH	Hepatic, choroidal metastasis	First-line use	Panic disorder Pleural effusion

MTC, medullary thyroid cancer; PTC, papillary thyroid cancer; NGS, next generation sequencing; LN, lymph node; VPC, ventricular premature contraction; PEG, percutaneous endoscopic gastrostomy; ICH, intracranial hemorrhage; ^a^ the adverse events are graded by Common Terminology Criteria for Adverse Events (CTCAE) from 1 to 4: Grade 1 (mild, G1), Grade 2 (moderate, G2), Grade 3 (severe, G3), Grade 4 (life-threatening, G4).

## Data Availability

The data presented in this study are available on request from the corresponding author. The data are not publicly available due to privacy or ethical restrictions.
